# Polypyrimidine tract binding protein knockdown reverses depression-like behaviors and cognition impairment in mice with lesioned cholinergic neurons

**DOI:** 10.3389/fnagi.2023.1174341

**Published:** 2023-04-27

**Authors:** Yiying Zhou, Ke Zhang, Fangmin Wang, Jiali Chen, Shanshan Chen, Manqing Wu, Miaojun Lai, Yisheng Zhang, Wenhua Zhou

**Affiliations:** ^1^Zhejiang Provincial Key Laboratory of Addiction Research, Ningbo Kangning Hospital, Health Science Center, Ningbo University, Ningbo, China; ^2^School of Life Sciences, Westlake University, Hangzhou, China; ^3^Department of Gynaecology and Obstetrics, Ningbo Medical Treatment Center, Affiliated Lihuili Hospital of Ningbo University, Ningbo, China; ^4^Shanghai Mental Health Center, Shanghai Jiao Tong University School of Medicine, Shanghai, China

**Keywords:** cognition function, depression, polypyrimidine tract binding protein, acetylcholine, astrocyte

## Abstract

**Background and objectives:**

Depression is a common comorbidity of dementia and may be a risk factor for dementia. Accumulating evidence has suggested that the cholinergic system plays a central role in dementia and depression, and the loss of cholinergic neurons is associated with memory decline in aging and Alzheimer’s patients. A specific loss of cholinergic neurons in the horizontal limb of the diagonal band of Broca (HDB) is correlated with depression and dysfunction of cognition in mice. In this study, we examined the potential regenerative mechanisms of knockdown the RNA-binding protein polypyrimidine tract binding protein (PTB) in reversing depression-like behaviors and cognition impairment in mice with lesioned cholinergic neurons.

**Methods:**

We lesioned cholinergic neurons in mice induced by injection of 192 IgG-saporin into HDB; then, we injected either antisense oligonucleotides or adeno-associated virus-shRNA (GFAP promoter) into the injured area of HDB to deplete PTB followed by a broad range of methodologies including behavioral examinations, Western blot, RT-qPCR and immunofluorescence.

**Results:**

We found that the conversion of astrocytes to newborn neurons by using antisense oligonucleotides on PTB in vitro, and depletion of PTB using either antisense oligonucleotides or adeno-associated virus-shRNA into the injured area of HDB could specifically transform astrocytes into cholinergic neurons. Meanwhile, knockdown of PTB by both approaches could relieve the depression-like behaviors shown by sucrose preference, forced swimming or tail-suspension tests, and alleviate cognitive impairment such as fear conditioning and novel object recognition in mice with lesioned cholinergic neurons.

**Conclusion:**

These findings suggest that supplementing cholinergic neurons after PTB knockdown may be a promising therapeutic strategy to revert depression-like behaviors and cognitive impairment.

## 1. Introduction

Multiple types of neurodegenerative diseases, such as Parkinson’s disease, Alzheimer’s disease (AD), and major depressive disorder (MDD), afflict the elderly, leading to a huge burden on public health (Rauf and Rahman, 2022; Suzuki et al., 2022). MDD has become the most common mental disease (Clevenger et al., 2018), manifests typically as a persistently low mood and lack of pleasure, often accompanied by cognitive impairment (Gong et al., 2023). At present, various antidepressants have been developed to improve a patient’s condition (Cipriani et al., 2009), and cognitive behavioral therapy has also been used (Wang et al., 2022). Since depressive symptoms of MDD are generally accompanied by cognitive impairment, we focused our attention on the basal forebrain (BF), which is closely related to cognition and emotion. Accumulating evidence suggests that a specific loss of cholinergic neurons, which is the innervation of the hippocampal function, is related to memory impairment and loss in aging and Alzheimer’s patients (Schliebs and Arendt, 1996).

The cholinergic system of the BF can be divided into different subpopulations (Semba, 2000). Neurons in the septum (MS) and Broca’s diagonal zone mainly provide cholinergic projections to the hippocampus and medial prefrontal cortex, while neurons in the large cell basal nucleus provide cholinergic innervation to the entire cortex and basolateral amygdala (Woolf, 1991; Amenta and Tayebati, 2008; Li et al., 2018). These cell populations control behavior by regulating the activity of the corresponding brain regions (Okada et al., 2021). Moreover, cholinergic neurons play an essential role in cognitive function. In aging process, downregulation of choline acetyltransferase (ChAT) activity and losing cholinergic neurons were observed in the brain, which was related to progressive memory deficits (Ferreira-Vieira et al., 2016). Thus, the destruction of cholinergic neurons has become a recognized method for constructing cognitive impairment models (Hernández-Melesio et al., 2019). Moreover, our previous results have shown that both depression and cognitive impairment could be produced by lesioning cholinergic neurons in the horizontal limb of the diagonal band of Broca (HDB) (Chen et al., 2021). Depression is a common comorbidity of dementia and may be a risk factor for dementia in patients with AD (Jorm, 2000). Therefore, it is reasonable to rescue cholinergic neurons to relieve depression-like behaviors and cognitive impairments.

In the acute stage of central nervous system injury, astrocytes begin to proliferate and express markers of neural stem/progenitor cells and neurogenic differentiation. The accumulating evidence indicates astrocytes were selected as the main candidate cells for transdifferentiation (Anderson et al., 2014; Magnusson et al., 2014). Recently, the reprogramming of glial cells into neurons *in vivo* has emerged as a promising method for treating a variety of neurodegenerative diseases (Guo et al., 2014; Liu et al., 2015; Brulet et al., 2017; di Val Cervo et al., 2017; Chen et al., 2020; Qian et al., 2020; Wu and Parry, 2020; Zhou et al., 2020; Xiang et al., 2021). Currently, astrocytes can be reprogrammed by regulating a single transcription factor (NeuroD1; (Chen et al., 2020) or polypyrimidine tract binding protein (PTB; Chen et al., 2020; Qian et al., 2020; Zhou et al., 2020)). PTB is an RNA-binding protein that plays a key role in the development of the nervous system (García-Blanco et al., 1989; Shibasaki et al., 1991). By knocking down this protein, transformation of astrocytes into functional neurons can be realized within a few weeks to a month (Qian et al., 2020; Zhou et al., 2020; Maimon et al., 2021). PTB knockdown in the substantia nigra (SN) can reprogram astrocytes, replenish lesioned dopaminergic neurons, and reverse the phenotypes of Parkinson’s mice (Qian et al., 2020). In mice with damaged retinas, PTB knockdown can replenish retinal ganglion cells and improve vision (Zhou et al., 2020). In aged mice, knockdown of PTB in the hippocampus can replenish pyramidal neurons and improve cognitive impairment (Maimon et al., 2021). In spinal cord injured mice, regional knockdown of PTB causes astrocytes to transform into motoneuron-like cells and restores motor function (Yang et al., 2023). However, direct reprogramming of astrocytes into cholinergic neurons in the HDB is still unclear.

Thus, we hypothesized that the number of lesioned cholinergic neurons will increase, the depression-like behaviors and cognitive impairment would improve, upon local knockdown of PTB. We used 192 IgG-saporin to inactivate ribosomes and prevent protein synthesis, leading to the loss of cholinergic neurons (Book et al., 1994). After lesioning cholinergic neurons, we used the forced swimming test (FST), tail suspension test (TST), and sucrose preference test (SPT) to detect depression-like behaviors. Moreover, we also performed locomotor activity test (LAT) to assess anxiety-related behaviors. In contrast, we used the novel object recognition test (NOR) and fear conditional test (FCT) to assess cognitive memory and learning or short-term memory deficits. First, we examined the efficacy of knocking down PTB using antisense oligonucleotides (ASOs) *in vitro* (Friedrich and Aigner, 2022; Le et al., 2022). Then, we administered a single injection of ASO-PTB or adeno-associated virus (AAVs)-shPTB into the lesioned area after injury of cholinergic neurons in HDB *in vivo* and investigated whether knockdown of PTB could transform astrocytes into newborn cholinergic neurons, relieve depression-like behaviors, and improve cognition function in lesioned cholinergic neurons of HDB.

## 2. Materials and methods

### 2.1. Animals

Adult male ICR mice (4 weeks old, *n* = 42, body weight, 25–30 g) were obtained from Zhejiang Provincial Experimental Animal Center (Hangzhou, China). Mice were housed in groups of five per standard mouse cage and were acclimated for at least 1 week prior to the start of the experiments. Mice were fed with food and water *ad libitum* and maintained with a reversed 12-h light/dark cycle (light onset at 8:30 PM, offset at 8:30 AM) in a temperature-and humidity-controlled room. All experimental procedures were approved by the Ethics Committee of Laboratory Animal Use and Care in Ningbo University (approval No. 11101) and also were accorded with the National Institutes of Health Guide for the Care and Use of Laboratory Animals (eighth edition).

### 2.2. Astrocytes transdifferentiation *in vitro*

Mouse astrocytes in cortical tissue was dissected from the whole brain from postnatal pups (P4–P5), digested by trypsin and plated on dishes coated with poly-d-lysine (Sigma, United States; Qian et al., 2020). Primary astrocytes were isolated and cultured in an astrocyte growth medium containing DMEM, 10% FBS, and penicillin/streptomycin (Gibco, United States). After reaching ~ 90% confluence, the cells were cultured in an astrocyte growth medium. The dishes were shaken overnight at 37°C to eliminate non-astrocytic cells. To analyze the effect of ASO-PTB on astrocytes *in vitro*, six-well plates of astrocytes were cultured and reached 70–80% confluency in an astrocyte growth medium, ASO-PTB (75 pmol per well) was transfected with Lipofectamine RNAimax (Thermo Fisher Scientific, United States). After ASO-PTB treatment for 48 h, the astrocytes were either collected for immunoblotting or continually cultured for 21 d (days).

### 2.3. Microinfusions

The anesthetized mice with sodium pentobarbital (50 mg/kg, *i.p.*) placed in a stereotaxic mouse frame. A microsyringe (Hamilton syringe, 5-μL) was inserted bilaterally into the HDB at following coordinates: an A/P (from bregma) = +0.74 mm, a M/L (from the midline) = ±0.625 mm, and a D/V (from the brain surface) = −4.75 mm (Paxinos, 2001). 192 IgG-saporin (Millipore, MA, United States) were injected per side in a volume of 0.25 μl with a concentration of 1 μg/μL as described before (Matchynski et al., 2013). The needle was slowly retracted 5 min after each injection. In all sham-operated mice, 0.25 μl) phosphate-buffered saline (PBS) was injected into both sides. All the liquids were injected at a rate of 0.1 μl/min. Following surgery, erythromycin ointment was applied to the affected area to prevent potential bacterial infection. The AAVs were injected at the lesion site (0.25 μl per injection) using the same microsyringe. The method for injecting ASOs (4 μg/μL, 0.5 μl per injection) was the same as that for injecting AAVs.

### 2.4. Synthesis of ASOs and AAVs

ASOs were synthesized by the Beijing Genomics institution. The ASO sequence for mouse PTB was 5′-GGGTGAAGATCCTGTTCAATA-3′. The all ASOs backbones contain modification with phosphorothioate. The target sequence of Turbo FITC was 5′-CAACAAGATGAAGAGCACCAA-3′. The 3′ end of ASOs was attached with Fluorescein for detection fluorescence. A small hairpin RNA (shRNA) targeting PTB was constructed and synthesized by BrainVTA Co., Ltd. (Wuhan, Hubei, China). The sequence of the shRNA used was as follows: 5′-GGGTGAAGATCCTGTTCAATA-3′ (AAV2/5, 2.31 × 10^12^ viral genomes VG/mL). The sequence of the shRNA (empty) used was as follows: 5′-CAACAAGATGAAGAGCACCAA-3′ (AAV2/5, 5.80 × 10^12^ VG/mL).

### 2.5. Experimental design

**Figure 1:**
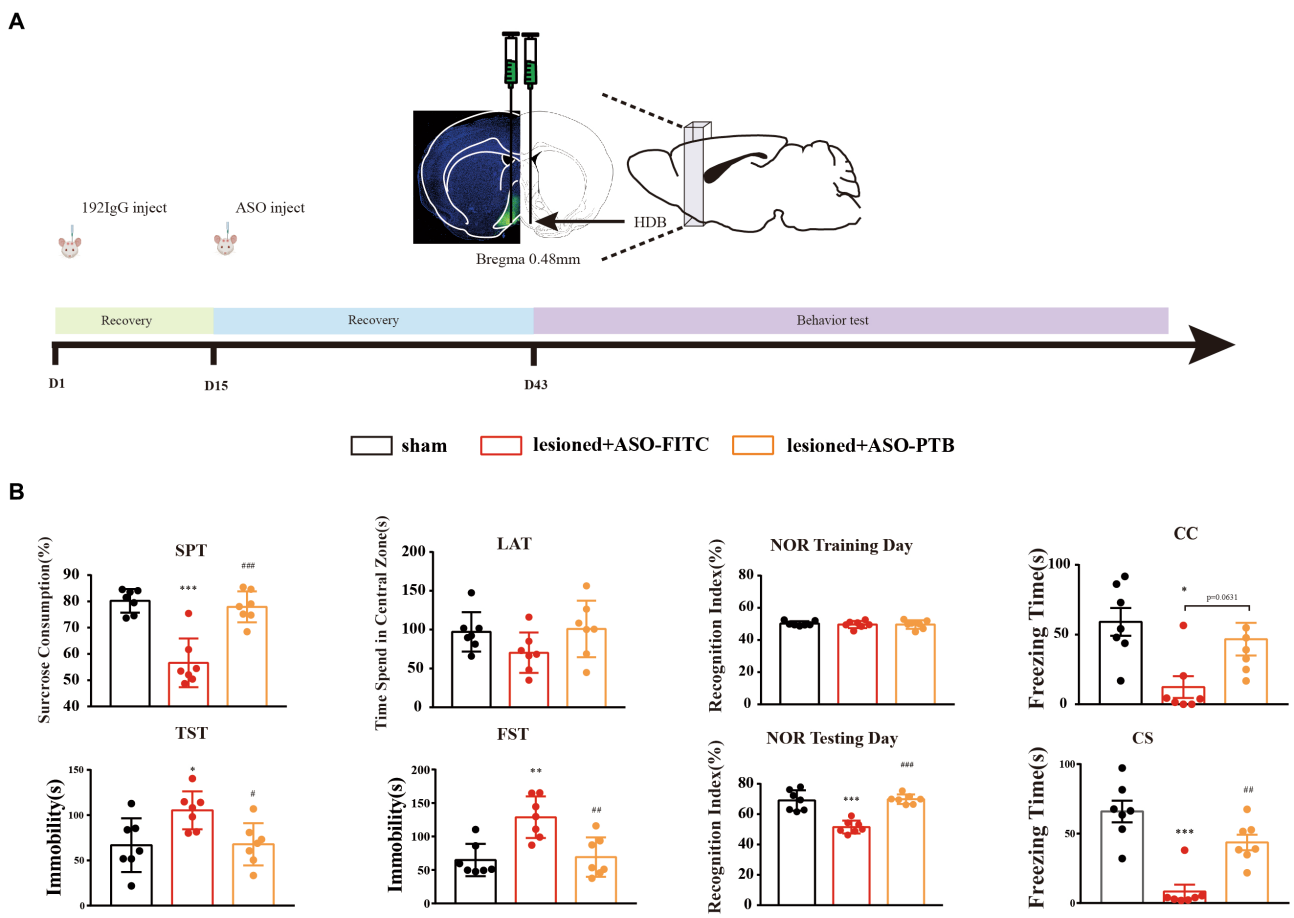
Conversion of astrocytes to neurons by ASO-PTB could relieve depressive-like behaviors and ameliorate cognition impairment phenotypes. **(A)** Experimental schedules. **(B)** The sucrose preference (one-way ANOVA, Tukey’s multiple comparisons test, *F* (2,18) = 25.26, ^***^p < 0.001, ^###^*p* < 0.001, *n* = 7); immobility time in the TST (one-way ANOVA, Tukey’s multiple comparisons test, *F* (2,18) = 5.42, ^*^p = 0.0254, ^#^*p* = 0.0293, *n* = 7) and FST (one-way ANOVA, Tukey’s multiple comparisons test, *F* (2,18) = 11.05 ^**^p = 0.0015, ^##^*p* = 0.0027, *n* = 7); the time spent in the central area in LAT (one-way ANOVA, Tukey’s multiple comparisons test, *F* (2,18) = 2.2, compared to the sham group, *p* = 0.2362, compared to the ASO-FITC group, *p* = 0.1615, *n* = 7); the cognitive memory about the time to explore novel object in NOR (one-way ANOVA, Tukey’s multiple comparisons test, *F* (2,18) = 30.76, ^***^p < 0.001, ^###^*p* < 0.001, *n* = 7); short-term memory deficit in contextual fear conditioning (CC) (One-way ANOVA, Tukey’s multiple comparisons test, *F* (2,18) = 5.893, ^**^p = 0.0103, ASO-FITC compared to the sham group, *p* = 0.0631, ASO-PTB compared to the ASO-FITC group, *n* = 7) and condition fear conditioning (CS) in FCT (one-way ANOVA, Tukey’s multiple comparisons test, *F* (2,18) = 21.73, ^***^p < 0.001, ^##^*p* = 0.0023, *n* = 7). ^*^Compared to the sham group, ^#^compared to the ASO-FITC group. The data are expressed as mean ± SEM.

**Figure 2:**
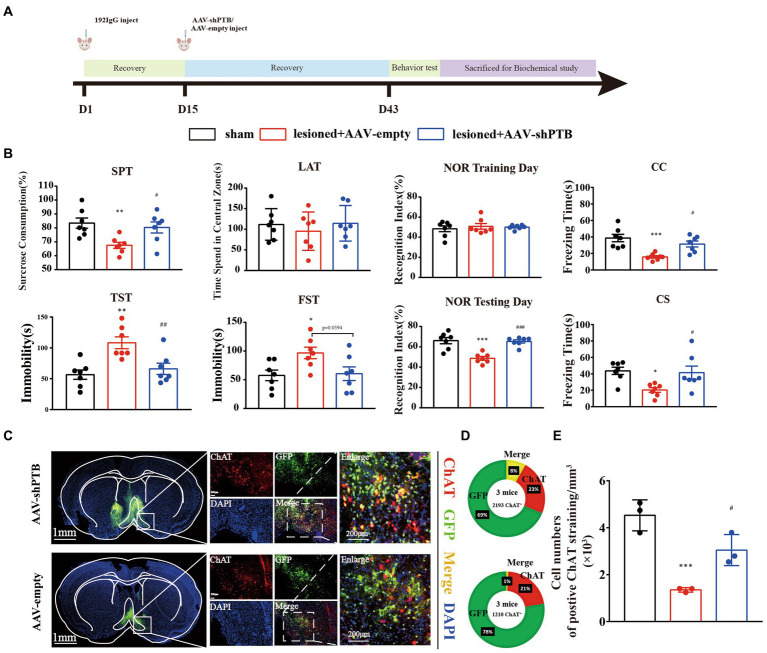
AAV-shRNA with GFAP promoters could replenish cholinergic neurons. **(A)** Experimental schedules. **(B)** The sucrose preference (one-way ANOVA, Tukey’s multiple comparisons test, *F* (2,18) = 6.235, ^**^p = 0.0099, ^#^*p* = 0.0389, *n* = 7); immobility time in the TST (one-way ANOVA, Tukey’s multiple comparisons test, *F* (2,18) = 9.712, ^**^p = 0.0017, ^##^*p* = 0.0089, *n* = 7) and FST (one-way ANOVA, Tukey’s multiple comparisons test, *F* (2,18) = 4.407, ^*^p = 0.0398, *p* = 0.0594 compared the AAV-empty group, *n* = 7); time spent in the central area in LAT (one-way ANOVA, Tukey’s multiple comparisons test, *F* (2,18) = 0.4025, *p* = 0.7607, compared to the sham group, *p* = 0.6898 compared to the AAV-empty group, *n* = 7); cognitive memory regarding the time to explore novel object in NOR (one-way ANOVA, Tukey’s multiple comparisons test, *F* (2,18) = 19.16, ^***^p < 0.001, ^###^*p* < 0.001, *n* = 7); and a short-term memory reversed in contextual fear conditioning (CC; one-way ANOVA, Tukey’s multiple comparisons test, *F* (2,18) = 11.36, ^***^p = 0.0005, ^#^*p* = 0.0135, *n* = 7) and conditional fear conditioning (CS) in FCT (one-way ANOVA, Tukey’s multiple comparisons test, *F* (2,18) = 5.351, ^*^p = 0.0215, ^#^*p* = 0.0387, *n* = 7). **(C)** Virus injection sites and co-labeling of cholinergic neurons with virus. Scale bar represents 1 mm (left); scale bar represents 200 μm (right). **(D)** The co-labeling rate of AAV-shRNA with green fluorescent protein and cholinergic neurons (top) and the co-labeling rate of AAV-empty with green fluorescent protein and cholinergic neurons (bottom). **(E)** The number of cholinergic neurons in AAV-PTB group was increasing significantly compared to the AAV-empty group, while the number of cholinergic neurons in AAV-empty group was increasing significantly compared to the sham group (one-way ANOVA, Tukey’s multiple comparisons test, *F* (2,6) = 25.73, ^***^p = 0.009, ^#^*p* = 0.0205). ^*^ Means compared to sham group, ^#^ means compared to AAV-empty group. Data are expressed as mean ± SEM.

Previously, we proved that cholinergic neurons loss in the HDB can produce depression and cognitive dysfunction. All behavioral tests were slightly modified based on previous studies (Bevins and Besheer, 2006; Castagné et al., 2011; Duclot et al., 2016; Chen et al., 2021). The experimental schedule is depicted in Figure 1A. Mice were divided randomly into three groups (*n* = 7 mice per group): Sham (sham operation), 192 IgG-saporin injection (ASO-FITC), and 192 IgG-saporin injection (ASO-PTB). Two weeks after 192 IgG-saporin injection, the mice injected with PBS (Sham), ASOs of FITC (ASO-FITC), and ASOs of PTB (ASO-PTB). Four weeks after surgery, the mice were subjected to six tests: SPT on d 43, TST on d 44, LAT on d 45, NOR on d 46, FST on d 48, and FCT on d 49. Experimental schedule for the subsequent protocol is depicted in Figure 2A. Mice were randomly divided into three groups (*n* = 7 mice per group): Sham-(sham operation), 192 IgG-saporin injection (AAV-empty), and 192 IgG-saporin injection (AAV-shPTB). Two weeks after surgery, the mice injected with PBS (Sham), AAV-empty (AAV-empty), and AAV-shPTB (AAV-shPTB). Four weeks after surgery, the mice were subjected to six tests in the SPT on d 43, TST on d44, LAT on d 45, NOR on d 46, FST on d 48, and FCT on d 49. Each behavioral test was conducted one trials. Behavioral tests were carried out between 1 and 7 PM. Before the test, the mice were placed in the test room for more than 2 h. All behavioral tests and data collection were blinded to the experimenters.

### 2.6. Sucrose preference test

Sucrose preference test was used to assess anhedonia (Müller et al., 2012). In the first 2 days, the mice were trained to adapt to 1% sucrose in individual cages. They were given one bottle of 1% sucrose solution and one bottle of tap water; their positions exchanged every 12 h. Then, deprivation of water and food for 24 h, the mice had free access to two bottles for another 24 h and measured both consumption volume. Sucrose preference (%) = sucrose consumption/(sucrose + water consumption) was calculated.

### 2.7. Forced swimming test

Forced swimming test was performed as described previously (Yankelevitch-Yahav et al., 2015). The mice were forced to swim individually in a cylinder (40 cm height, 15 cm diameter). The cylinders were filled with tap water at 24 ± 0.5°C. The water depth was adjusted according to the mouse’s size, so that it could not touch the bottom of the container with its hind legs. The mice were gently placed into the water and recorded with a camera for 6 min. The tested mouse was then removed and dried with a towel, following which water in the bucket was replaced to test the next mouse.

### 2.8. Tail-suspension test

Tail-suspension test was carried out as described previously (Lv et al., 2012), with minor modification. The TST apparatus consisted of a shelf (25 cm distance from floor). The mouse tail tip was fixed by approximately 1 cm on the shelf with an adhesive tape. The mice were suspended for 6 min, and the static time was recorded in the last 4 min.

### 2.9. Locomotor activity test

Locomotor activity test was performed as described previously (Xu et al., 2017). Each mouse was placed in a 100 × 100 × 40 cm^3^ open-field chamber and allowed to explore it freely for 10 min, with a camera recording its activity. Horizontal distance was recorded by using infrared photobeam sensors and analyzed using an ANY-maze behavioral recording system (Stoelting, United Kingdom). Movement time was measured when a mouse was active for > 1 s. The chamber was wiped with 75% ethanol solution after each test.

### 2.10. Fear conditioning test

Fear conditioning test is a paradigm used to evaluate fear memory and learning performance (Wilensky et al., 2000). Each mouse was placed in a chamber [40 (L) × 40 (W) × 50 (H) cm^3^] with a grid floor and allowed to explore it freely for 2 min on the day before the testing day. Then, a conditioned stimulus (30s, 4 kHz, 90 dB), followed by an electric foot-shock (2S, 0.6 mA), was applied. The presentation of the conditioned stimulus was repeated five times at 60 s intervals. The animals were put to their home cage at 30 s after the last shock immediately. After 24 h, the mice were put back to the previous chambers without any tone or foot shock (CC) for 3 min, then tested with tone (CS) for 3 min. The context-and condition-dependent freezing times of each mouse were recorded and analyzed using video freeze software (Med Associates, United States).

### 2.11. Novel object recognition

This test was performed on day 46 and 47. On the first day, the animals were allowed to explore two identical objects [black cubes: 5.5 (L) × 5.5(W) × 5.5 (H) cm] for 5 min. On the second day, the animals were placed in the same cage with a familiar object and a novel object. The time spent with the familiar object and novel object [white pyramid: 8 (L) × 5.5(W) × 8 (H) cm] was noted. The open area box and objects were cleaned with 75% ethanol solution after each test (Zhang et al., 2012).

### 2.12. Brain tissue fixation

The mice were deeply anesthetized with sodium pentobarbital (50 mg/kg, *i.p*.) after complement of all behavioral tests, and perfused routine with sterile saline followed by 4% paraformaldehyde solution (PFA) in 0.1 M phosphate buffer (PB, pH 7.4) through the heart. The mouse brains were post-fixed with PFA at 4°C overnight, then kept for further use in 30% sucrose in 0.1 M PB solution at 4°C.

### 2.13. Western blotting

Western blotting was performed as described previously (Zhu et al., 2021). Briefly, cells or brain tissues were extracted at 4°C for 1 min using lysis buffer and centrifuged at 16000 *× g* for 10 min. The protein levels in the supernatant were estimated using Bradford assay, followed by SDS-PAGE of tissue samples (40 μg) and subsequent transfer of protein bands to polyvinylidene fluoride membranes. The membranes were blocked with 5% non-fat milk in TBST for 2 h and incubated overnight at 4°C with primary antibodies against PTB (1:500, A6107, Abclonal, Wuhan, China) and β-actin (1:5000, ABclonal, AC026, Wuhan, China). After washing the samples three times with TBST, the membranes were incubated with an Alexa Fluor 800 conjugated secondary antibody (1:5000) for 60 min. Detection and quantification of specific bands were performed using a fluorescence scanner (Odyssey Infrared Imaging System, LI-COR Biotechnology, Lincoln, NE, United States). All the data are representative of three independent experiments and were expressed as ratios of optical density (OD) of samples to that of controls for statistical analyses.

### 2.14. Quantitative reverse transcription polymerase chain reaction (RT–qPCR)

The mice were sacrificed for RT-qPCR analysis. Total RNA was extracted from HDB cells using TRIzol reagent (Thermo Fisher, Waltham, MA, United States; Zhang et al., 2021). Glyceraldehyde-3-phosphate dehydrogenase (GADPH) and β-actin mRNA levels were used as controls. Relative mRNA expression was calculated using the 2^–ΔΔCT^ method. The primer sequences were as follows:

**Table 1:** 

PTB	F:AGCCAATGGAAACGATAGCAA R:GCGCCACCGATGTATAGTAGT
GAPDH	F: CATGGCCTTCCGTGTTCCTA; R: TACTTGGCAGGTTTCTCCAGG.
β-actin	F:CCTAAGAGGAGGATGGTCGC R:CTCAGACCTGGGCCATTCAG

### 2.15. Immunofluorescence

Total neurons and cholinergic neurons in the HDB were assessed by using NeuN and choline acetyltransferase (ChAT) immunofluorescence. Briefly, the coronal brain sections at 30-μm thick were cut using a cryostat (Leica CM1950, Heerbrugg, Switzerland). The floating sections were rinsed with PBS, blocked with BSA (1% in PBS) and 0.2% Triton X-100 for 1 h at room temperature, and then incubated in a primary antibody solution containing rabbit polyclonal NeuN (1:500; 94,403; CST, Danvers, MA, United States) and ChAT (1:1000; ab178850; Abcam, Cambridge, United Kingdom) in blocking buffer for 12 h at 4°C. After three times with PBS washing, the sections were incubated with secondary goat antibodies [anti-rabbit IgG (1:500; A11012; Invitrogen, United States) mixed with FITC goat anti-mouse IgG (H + L; 1:300; AS001; Abclonal, Wuhan, China)] for 2 h at 37°C. Sections were washed thoroughly with PBS (1×) thoroughly for 1 h and mounting was performed with antifading medium (ab104139, Abcam, Cambridge, United Kingdom).

Mice injected with viruses containing the GFAP promoter in the HDB were sacrificed and processed for anti-GFAP immunofluorescence. The primary antibody used was mouse polyclonal GFAP (1:1000; 3,670; CST, Danvers, MA, United States). Mouse primary astrocytes transfected with ASO-PTB were stained with a doublecortin antibody (1:1000; 4,604; CST, Danvers, MA, United States). The other operation steps were the same as those described above. All fluorescent images were captured using a BX41 microscope (Olympus, Tokyo, Japan) or a laser confocal microscope (Leica, Heerbrugg, Switzerland). Immunofluorescence *via* digital image analysis was quantified by using the Image-Pro Plus 6.0 software.

### 2.16. Statistical analysis

The number (*n*) of replicated samples or mice is listed in individual figure legends. The experiments were not randomized, and no statistical methods were used to predetermine the sample size. Experimental variations in the graph are presented as the mean ± standard error of the mean (SEM). All measurements were performed using independent samples. Independent t-tests and one-way ANOVA for statistical analyses are indicated in the individual figure legends. The data was analyzed by using GraphPad Prism§ v7.0 (GraphPad Software, CA, United States), and the accepted significance value for all tests was *p* < 0.05.

## 3. Results

### 3.1. Conversion of astrocytes to immature neurons by ASO-PTB *in vitro*

**Figure 3:**
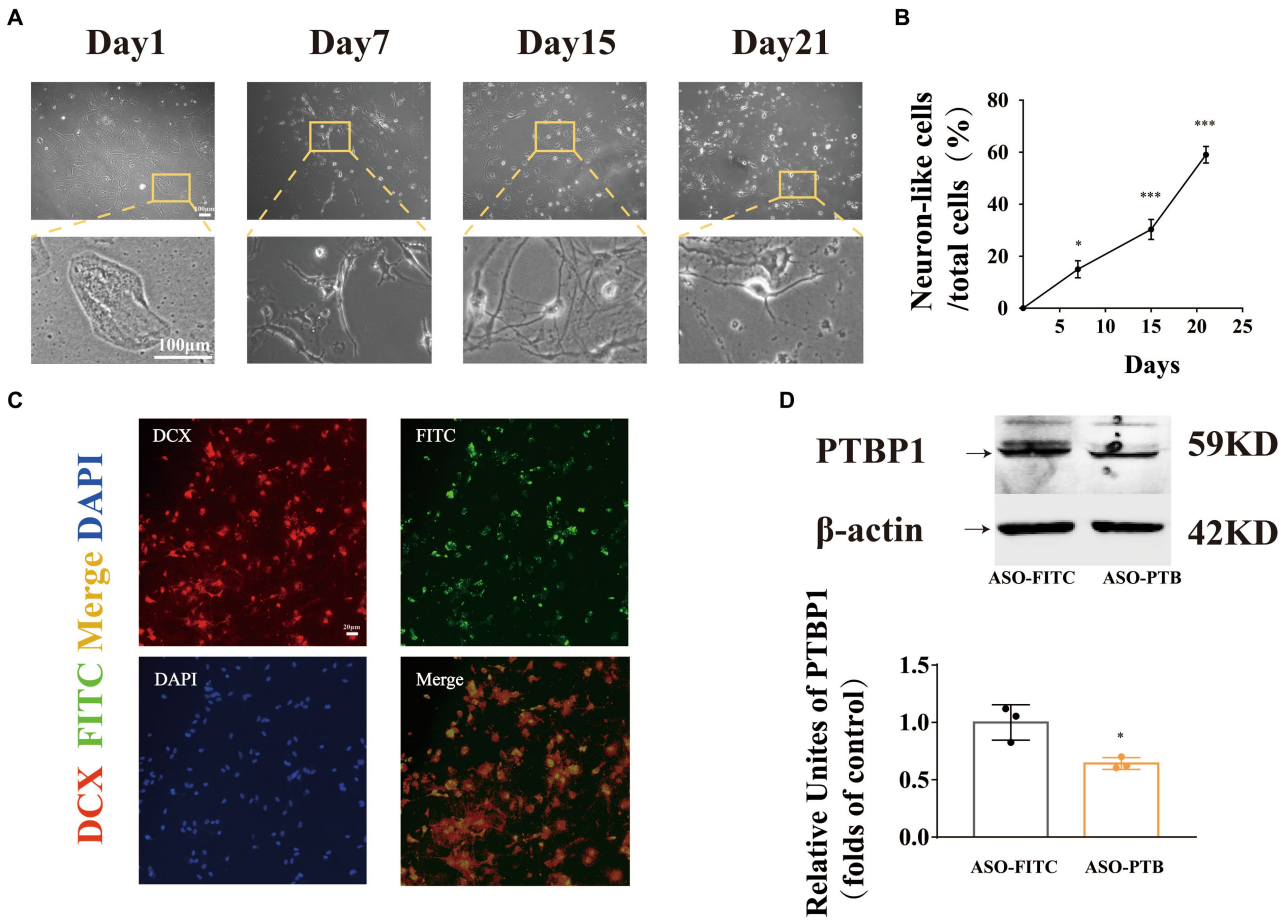
Conversion of astrocytes to immature neurons by ASO-PTB *in vitro*. **(A)** Mouse primary astrocytes transform into neuron-like cells. Original objective magnification of 4 × (top), the images below are an enlarged image (bottom), Scale bar represents 100 μm. **(B)** The length of axons and the number of neuron-like cells increased following incubation (one-way ANOVA, Tukey’s multiple comparisons test, *F* (3,8) = 120.3 ^*^p = 0.0312, ^***^p = 0.004, ^***^p < 0.001 compared to the first day, *n* = 3). **(C)** Cortical astrocytes, treated with ASO-PTB, were stained for doublecortin (red) and DAPI (blue); FITC was carried by ASO (green). Scale bar represents 20 μm. **(D)** Western blotting and quantification of PTB knockdown in mouse Primary astrocytes (two-tailed unpaired *t*-test, *t* = 3.819, *df* = 4, ^*^p = 0.0188 compared to the ASO-FITC, *n* = 3). The arrow refers to the target band. Data are expressed as mean ± SEM.

To prove that PTB knockdown can transform mouse primary astrocytes into neurons, we transferred ASO-PTB into astrocytes using Lipofectamine. Three weeks later, astrocytes showed neuronal morphology (Figure 3A). The length of axons and neuron-like cells increased over time (Figure 3B). Subsequently, we stained the cells with the newborn neuron marker doublecortin (DCX). We found that most astrocytes transfected with ASO-PTB were DCX-positive (Figure 3C). After transfer to ASO-PTB for 48 h, the protein content of PTB in ASO-PTB-treated cells decreased to 60% (Figure 3D).

### 3.2. Conversion of astrocytes to neurons by ASO-PTB in HDB-cholinergic lesioned mice

**Figure 4:**
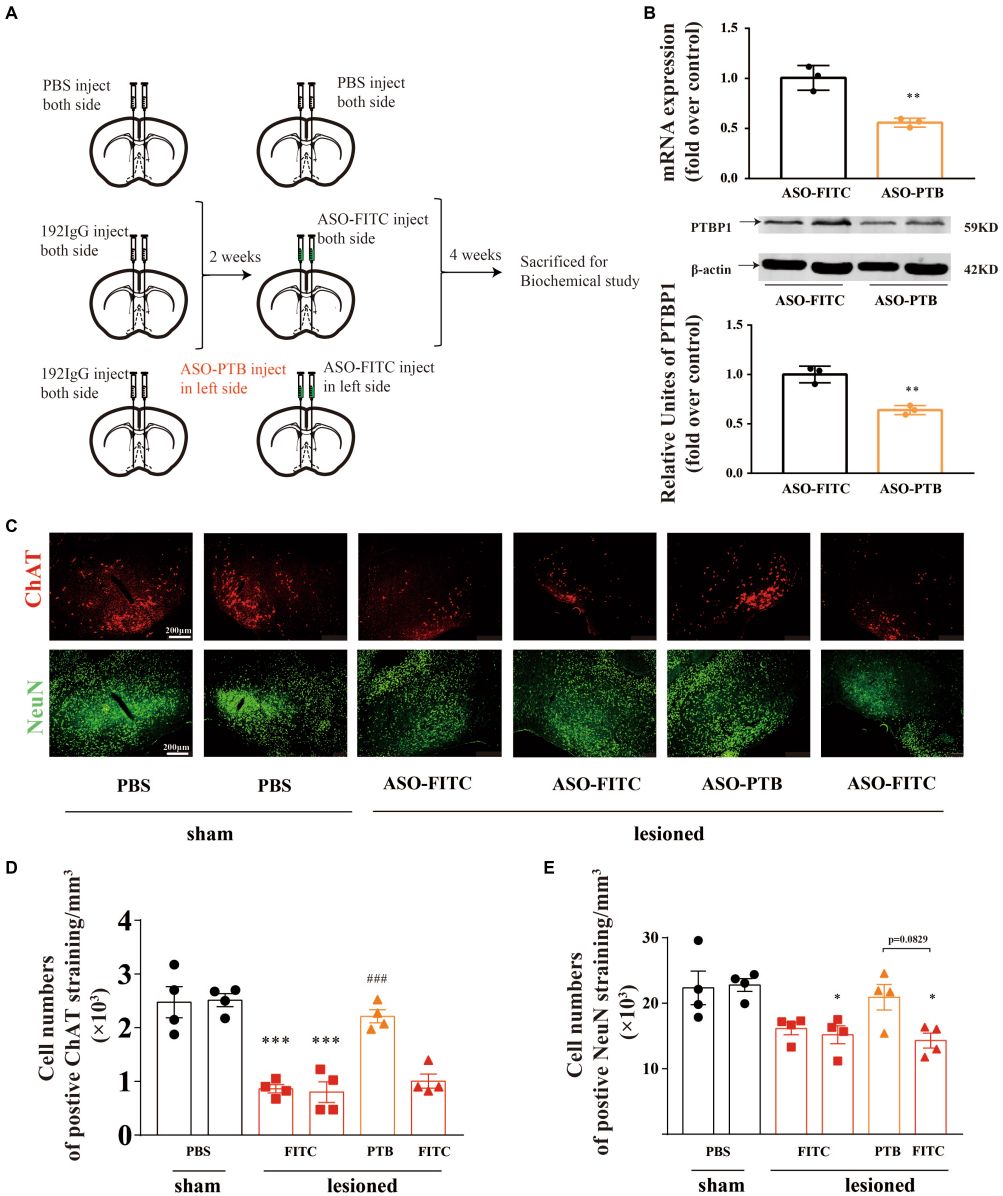
Conversion of astrocytes to neurons by ASO-PTB in HDB-cholinergic lesioned mice. **(A)** Experimental schedules. **(B)** The mRNA (two-tailed unpaired *t*-test, *t* = 5.268, *df* = 4, ^**^p = 0.0062, *n* = 3) and protein (two-tailed unpaired *t*-test, *t* = 6.472, *df* = 4, ^**^p = 0.0029, *n* = 3) contents of PTB in mice were significantly decreased by injecting ASO-PTB, compared to the ASO-FITC group. The arrow refers to the target band. **(C)** HDB brain area, co-stained with ChAT (red) and NeuN (green). Scale bar represents 200 μm. **(D)** Significant differences in density of ChAT-positive cell between the sham and 192 IgG-saporin groups by quantitative analysis. ASO-PTB injection significantly increased the number of cholinergic neurons (one-way ANOVA, Tukey’s multiple comparisons test, *F* (5,18) = 24.16, ^***^p < 0.001, from left to right, compared to the sham site, ^###^*p* < 0.001, compared to the ASO-FITC, *n* = 4). **(E)** Significant increase in density of NeuN-positive cell between the sham and 192 IgG-saporin groups. ASOs injection significantly increased the number of neurons (one-way ANOVA, Tukey’s multiple comparisons test, *F* (5,18) = 5.703, ^*^p = 0.0351, ^*^p = 0.0234, from left to right, compared to the sham left site, *p* = 0.0829 compared to the lesioned right site, *n* = 4). ^*^Compared to the sham group, ^#^compared to the ASO-FITC group. Data are expressed as mean ± SEM.

After verifying that astrocytes have the potential to transform into neurons, we destroyed cholinergic neurons by injecting 192 IgG-saporin into both sides of the HDBs of the mice. Two weeks later, we injected ASOs of PTB (ASO-PTB) into the left side of HDB to knockdown PTB and injected ASOs of FITC (ASO-FITC) into the right side of HDB (Figure 4A). After 4 weeks, immunofluorescence staining was performed for ChAT and NeuN (Figure 4C). We found that the number of cholinergic neurons (ChAT-positive) in the left HDB (ASO-PTB-treated side) was significantly higher than that in the right HDB and both sides HDB (ASO-FITC; Figure 4D). And the number of NeuN-positive neurons in ASO-PTB-treated side increased compared to ASO-FITC-treated side (*p* = 0.0829; Figure 4E). We also proved that the injection of ASO-PTB can knock down PTB at both the RNA and protein levels (Figure 4B).

### 3.3. Conversion of astrocytes to neurons by ASO-PTB could relieve depressive-like behaviors and ameliorate cognition impairment phenotypes

We continued to validate the therapeutic potential of ASO-PTB in depressive-like behaviors and cognitive impairment phenotypes after lesion of cholinergic neurons by 192 IgG-saporin for 2 weeks. Four weeks after injecting ASO-PTB into both sides of the HDB, depression and cognitive behavioral tests were performed (Figure 1A). The effect of ASO-PTB injection on depression-like behaviors induced by HDB-cholinergic lesions in mice is shown in Figure 1B. SPT results decreased significantly in the ASO-FITC group, compared to the sham group. In the ASO-PTB group, SPT results increased significantly, compared to the ASO-FITC group, suggesting that damaging cholinergic neurons leads to anhedonia in mice and can be reversed by replenishing cholinergic neurons; similarly, in the FST and TST results, immobility time increased significantly in the ASO-FITC group, compared to the sham group. In the ASO-PTB group, it decreased significantly, compared to the ASO-FITC group, suggesting that damaging cholinergic neurons leads to hopelessness in mice and can be reversed by replenishing cholinergic neurons; in the LAT, the time spent in the center area in the ASO-FITC group decreased compared to the sham group. In the ASO-PTB group, the time spent in the central area increased compared to that in the ASO-FITC group. However, there was no difference in the total distance between every group of mice, suggesting that the injection of ASO-PTB into the HDB failed to alter the locomotor activity of the mice (Additional Figure 1A). NOR showed that the time to explore novel objects significantly decreased in the ASO-FITC group compared to that in the sham group. In the ASO-PTB group, the time to explore novel objects increased compared to that in the ASO-FITC group, suggesting that replenishment of cholinergic neurons can reverse cognitive dysfunction. Moreover, there were significant differences in the freezing time among the groups. The ASO-FITC group exhibited learning or short-term memory deficits in fear conditioning. In the ASO-PTB group, learning or short-term memory deficits improved compared to those in the ASO-FITC group (Figure 1B).

### 3.4. Adeno-associated virus-shRNA knock down PTB by degrading PTB mRNA

**Figure 5:**
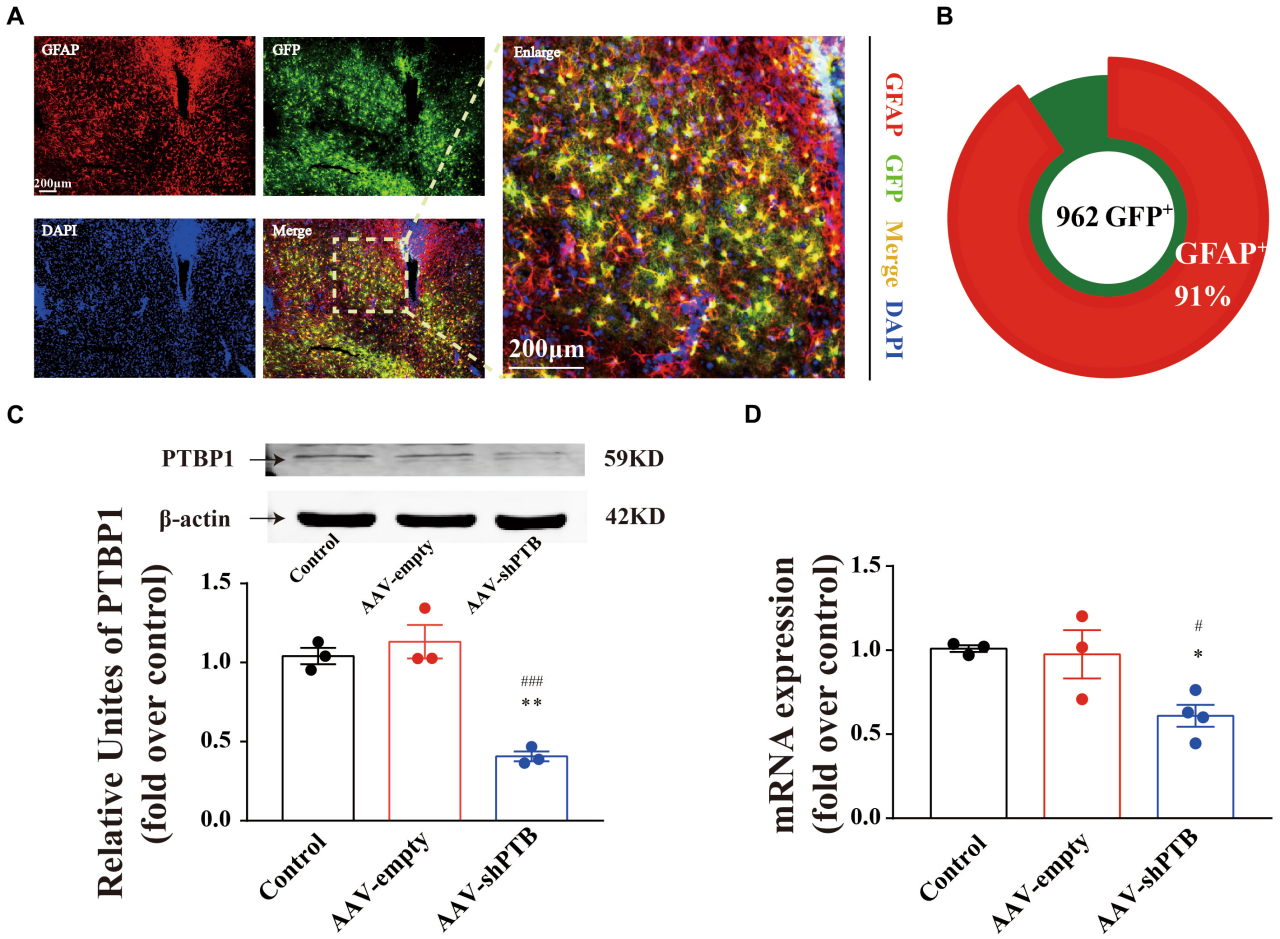
Adeno-associated virus-shRNA knock down PTB by degrading PTB mRNA. **(A)** Co-labeling of astrocyte promoter virus and GFAP protein. The images in right are an enlarged image. Scale bar represents 200 μm. **(B)** The co-labeling rate of AAV with GFAP promoter and astrocytes was 91%. **(C,D)** The protein (one-way ANOVA, Tukey’s multiple comparisons test, *F* (2,6) = 31.56, ^**^p = 0.0017, ^###^*p* = 0.0008, *n* = 3) and mRNA (one-way ANOVA, Tukey’s multiple comparisons test, *F* (2,7) = 6.959, ^*^p = 0.0317, ^#^*p* = 0.0460, *n* = 3 in sham and AAV-empty group, *n* = 4 in AAV-shPTB group) contents of PTB in mice were significantly decreased by injecting AAV-shPTB. The arrow refers to the target band. ^*^Means compared to sham group, ^#^means compared to AAV-empty. The data are expressed as mean ± SEM.

To verify the specificity of the GFAP promoter, we demonstrated the specificity of the virus by co-labeling GFAP protein with the astrocyte promoter, with a co-labeling rate of 91% (Figures 5A,B). Using RT-qPCR and western blotting, we proved that the injection of AAV-shRNA can knock down PTB at both the RNA and protein levels (Figures 5C,D).

### 3.5. Adeno-associated virus-shRNA with GFAP promoters could replenish cholinergic neurons

We have demonstrated an increase in the number of cholinergic neurons but did not prove that neurons are converted from astrocytes. Therefore, after destroying cholinergic neurons, we injected an AAVs with the GFAP promoter. Four weeks after AAVs injection, we performed the same behavioral tests on the mice (Figure 2A). Effects of ASO-PTB injection on depression-like behaviors induced by HDB cholinergic lesions in mice are shown in Figure 2B. SPT results decreased significantly in the AAV-empty group, compared to the sham group. In addition, the AAV-shPTB group showed a significant increase compared to the AAV-empty group, suggesting that the replenishment of cholinergic neurons can reverse anhedonia in mice. Similarly, there were significant differences in immobility time in the FST and TST between the AAV-empty and sham groups. In the AAV-shPTB group, it decreased significantly compared to the AAV-empty group, suggesting that replenishment of cholinergic neurons can reverse hopelessness in mice. The time spent in the center area decreased in the AAV-empty group compared to the sham group in LAT. In the AAV-shPTB group, the time spent in the central area increased compared to that in the AAV-empty group, but, the difference was not statistically significant. In addition, there was no difference in the total distance among the groups (Additional Figure 1B). The NOR group showed a significant cognitive memory deficit in the AAV-empty group compared with the sham group. In the AAV-shPTB group, cognitive memory improved compared to that in the AAV-empty group. Moreover, there were significant differences in the freezing time among the groups. The AAV-empty group had learning or short-term memory deficits in fear conditioning. In the AAV-shPTB group, learning or short-term memory deficits improved compared to those in the AAV-empty group (Figure 2B). Subsequently, we stained the brain tissue of mice and found that the co-labeling rate of AAV-shRNA with green fluorescent protein and cholinergic neurons reached 8%. The concern that AAV-shPTB might infect some endogenous ChAT neurons as the GFAP promoter may have a degree of leaky expression in neurons, we further examined the AAV-empty (which expresses GFP but not shPTB) with neurons. Few GFP+ cells stained positively for ChAT in the HDB of mice treated with AAV-empty was observed compared with AAV-shPTB-treated mice, as quantified on the bottom. The co-labeling rate of negative viruses (only 1%) was significantly lower than that of AAV-shPTB (8%; Figures 2C,D). The number of cholinergic neurons also increased significantly in AAV-shPTB group (Figure 2E).

## 4. Discussion

The main findings are that primary astrocytes transferred by ASO-PTB *in vitro* exhibited the neuron-like morphology and showed double cotin (DCX) positive. *In vivo*, we injected ASO-PTB into HDB in cholinergic neuron-lesioned mice and found that the number of cholinergic neurons increased significantly in the ASO-PTB group, compared to the ASO-FITC group. Depressive-like behaviors and cognitive impairments were also reversed. Subsequently, we constructed AAV-shPTB with the GFAP promoter to knock down PTB in astrocytes. The number of cholinergic neurons increased, depressive-like behaviors were relieved, and cognition impairment was alleviated in mice after knockdown of PTB by AAV-shPTB. The results demonstrated that astrocytes transform into cholinergic neurons *in situ* by knocking down PTB and can improve depression-like behaviors and cognitive impairment by replenishing the lesioned cholinergic neurons *via* PTB knockdown.

ASO-PTB is a synthetic, chemically modified, single strand of 21 nucleotides. It can combine with mRNA and recruit RNase to degrade mRNA and inhibit PTB protein translation (Suzuki et al., 2022). In the present study, primary astrocytes exhibited DCX^+^ cells colocalized with FITC green fluorescent protein carried by ASO-PTB and ASO-PTB can knock down PTB at the mRNA and protein levels (Qian et al., 2020), which is consistent with the findings of Wang et al. who found DCX + after over expression NEUROD1 in the U251 cell line (Couillard-Despres et al., 2005; Wang et al., 2021). Neurons are mature cells with no regenerative capacity (Zhao et al., 2008; Lei et al., 2019). Astrocytes are common target cells for neuronal transformation because their activation status in neurodegenerative diseases and stroke can lead to disease pathology (Vasan et al., 2021; Bocchi et al., 2022), and transdifferentiation of astrocytes into neurons or reprogramming into neuroblasts have been reported (Heinrich et al., 2010; Niu et al., 2013; Torper et al., 2013). For example, it can transform reactive astrocytes into functional neurons through PTB downregulation (Qian et al., 2020) or overexpression of either SOX2 or NeuroD1(Chen et al., 2020; Tai et al., 2021). In the present study, 192 IgG-saporin penetrates into the cell and results in the loss of cholinergic neurons (Chen et al., 2021). We then injected ASO-PTB into one side of the HDB and ASO-FITC into the other side of the HDB to make a more intuitive comparison of the ASO-PTB effect on neurons transformed *in situ*. The present results showed that the number of cholinergic neurons in the ASO-PTB-treated side increased compared to that in the ASO-FITC side, indicating that knockdown of PTB could induce the trans-differentiation of astrocytes into cholinergic neurons.

AAV with specific promoters have been designed to specifically bind to target cell populations. Considering the lack of cell specificity for ASOs, AAVs with the GFAP promoter was used in the present study. We confirmed that the co-labeling rate of the GFAP promoter AAVs with astrocytes was 91% after the virus was injected to HDB. In addition, we stained the HDB site with ChAT and observed co-labeling of GFP carried by AAVs with ChAT-positive neurons. The co-labeling rate of AAV-shRNA with GFP and cholinergic neurons reached 8%, while only 1% of AAV-empty co-labeling neurons. However, AAVs has the problem of leakage, and Wang et al. raised a challenge in that transformed neurons may not be transformed from astrocytes using stringent lineage-tracing strategies. Recently, Xu et al. proposed that AAVs vectors should be kept in a titer range of 10^11^–10^12^ GC/mL when injected into healthy mouse brains. The AAVs vector GFAP::GFP starts to express GFP in neuronal cells when the AAVs titer reaches 10^13^ GC/mL, breaking down the restriction of the GFAP promoter and leading to “neuronal leakage” (Xu et al., 2022). In our research, we used AAV2/5 at 2.31 × 10^12^ viral genomes (VG)/mL (0.25 μl per side) to knock down PTB, indicated that increasing ChAT neurons by knockdown PTB might not be due to the leakage of AAVs vectors.

Cholinergic neuron loss was observed in HDB after the administration of 192 IgG-saporin (Chen et al., 2021). Neuronal atrophy in the rodent hippocampus and prefrontal cortex caused by exposure for chronic stress leads to produce of depressive-like behaviors (Banasr et al., 2007). The special lesions of cholinergic neurons is accompanied by astrocytes activation and microglial proliferation in the hippocampus (Dobryakova et al., 2019). Elevated levels of proinflammatory cytokines followed activated microglia and astrocytes may account for the pathogenesis of depression after HDB damage (Singhal and Baune, 2017). In the present study, the lesioned mice showed anhedonia in the SPT and hopelessness in the FST and TST. The cognitive memory regarding the time spent for novel objects decreased, and short-term memory deficits in contextual and conditional fear conditioning were observed. These depression-like behaviors and cognitive impairment were reversed by PTB knockdown induced by injecting either ASO-PTB or AAV-shPTB into the lesioned area after injury of cholinergic neurons in HDB *in vivo*. This improvement was parallel to the transformation of astrocytes into newborn cholinergic neurons in the HDB. In addition, in the LAT, there was no difference in the total distance between the groups, suggesting that this improvement did not account for the locomotor activity of mice. The results demonstrated that astrocytes transform into cholinergic neurons *in situ* by knocking down PTB in cholinergic neuron lesions, which can relieve depression-like behaviors and alleviate cognitive impairment.

Existing experimental results suggest that in different brain regions, astrocytes have different transcriptional and proteomic environments, as well as region-specific neural transcription factors, which lead to the transdifferentiation of astrocytes into different subtype-specific neurons after reprogramming (Mattugini et al., 2019; Herrero-Navarro et al., 2021; Kempf et al., 2021). For example, there are mainly dopaminergic neurons in the SN. Therefore, astrocytes are mostly transformed into dopaminergic neurons, rather than other cells (Qian et al., 2020). The hippocampus is mainly composed of pyramidal neurons; therefore, in the study by Maimon et al. (2021), astrocytes were also mainly transformed into glutamatergic neurons. In addition, Yang et al. reported that PTB knockdown can increase the number of cholinergic neurons in the injured spinal cord. Cholinergic neurons are mainly distributed in the HDB and project to various brain regions. Therefore, after PTB knockdown, we found that most newborn neurons in HDB were cholinergic neurons. These results indicated that the transformation of astrocytes into different subtype-specific neurons depends on their environment.

As ASOs are simple to manufacture and easy to adjust, they are considered to have great clinical therapeutic potential (Huang et al., 2022). ASO-PTB can knock down PTB at the mRNA and protein levels. It is also feasible to use AAV as carriers in clinical practice. Currently, the therapeutic method of transforming coagulation factor IX (FIX) expression cassette into liver cells through an AAVs vector has been approved (Berntorp and Shapiro, 2012). Compared with ASOs, AAVs has a lower probability of mistargeting and causes less inflammatory reactions in the body (Rao et al., 2009). Additionally, AAVs has thermostability, good tolerance, and durable immunogenicity, making it feasible for use as a carrier in clinical practice (Zabaleta et al., 2021). Moreover, AAVs with specific promoters have been designed to specifically bind to target cell populations. ASO-PTB or AAV-shPTB, as gene therapeutics to knock down PTB, may have potential use in the treatment of depression and dementia in clinical settings. Direct reprogramming of endogenous glial cells into functional neurons provides a new opportunity for nerve-regeneration (Wang et al., 2021). Based on current research, the increase in number of neurons may have occurred *via* transformation from astrocytes or differentiation from residual neural stem cells, owing to PTB knockdown. The current study has some limitations, such as the failure to verify the function of neurons by *in vitro* electrophysiology and whether newborn cholinergic neurons project to corresponding brain regions to perform their functions. Moreover, the sex of the animal used had not been considered. Many studies have shown the gender differences in the depression (Sanchez et al., 2018) and in the pharmacological effects on the cholinergic stimulation (Giacobini and Pepeu, 2018), thus, the generalizability of these results to females is not obvious.

## 5. Conclusion

The results demonstrated that knockdown of PTB in HDB by ASOs or AAVs could specifically transform astrocytes into cholinergic neurons, then relieve the depression-like behaviors and protect against memory loss induced by cholinergic neuron lesions in HDB, suggesting that generating programmed cholinergic neurons following PTB knockdown may be a promising therapeutic strategy to improve the comorbidity statistics associated with depression and dementia.

## Data availability statement

The original contributions presented in the study are included in the article/Supplementary material, further inquiries can be directed to the corresponding authors.

## Ethics statement

The animal study was reviewed and approved by Ethics Committee of Laboratory Animal Use and Care in Ningbo University.

## Author contributions

YYZ and KZ performed research, analysis the data, and the writing of the paper. FW, JC, SC, and MW performed research. ML were responsible for guarantee of animal and experimental conditions. YSZ and WZ were responsible for the study design and revising the paper. All authors contributed to the article and approved the submitted version.

## Funding

This work was supported by Natural Science Foundation of China (82071499), Zhejiang Medical and Health Leading Academic Discipline Project (00-F06), and Innovation project of distinguished medical team in Ningbo (2022030410).

## Conflict of interest

The authors declare that the research was conducted in the absence of any commercial or financial relationships that could be construed as a potential conflict of interest.

## Publisher’s note

All claims expressed in this article are solely those of the authors and do not necessarily represent those of their affiliated organizations, or those of the publisher, the editors and the reviewers. Any product that may be evaluated in this article, or claim that may be made by its manufacturer, is not guaranteed or endorsed by the publisher.

## Supplementary material

The Supplementary material for this article can be found online at: https://www.frontiersin.org/articles/10.3389/fnagi.2023.1174341/full#supplementary-material
