# Supplementary figures and images for: Polypyrimidine tract binding protein knockdown reverses depression-like behaviors and cognition impairment in mice with lesioned cholinergic neurons
Source: Front Aging Neurosci. 2023 Apr 27;15:1174341. doi: 10.3389/fnagi.2023.1174341 (PMC10172502; doi:10.3389/fnagi.2023.1174341)

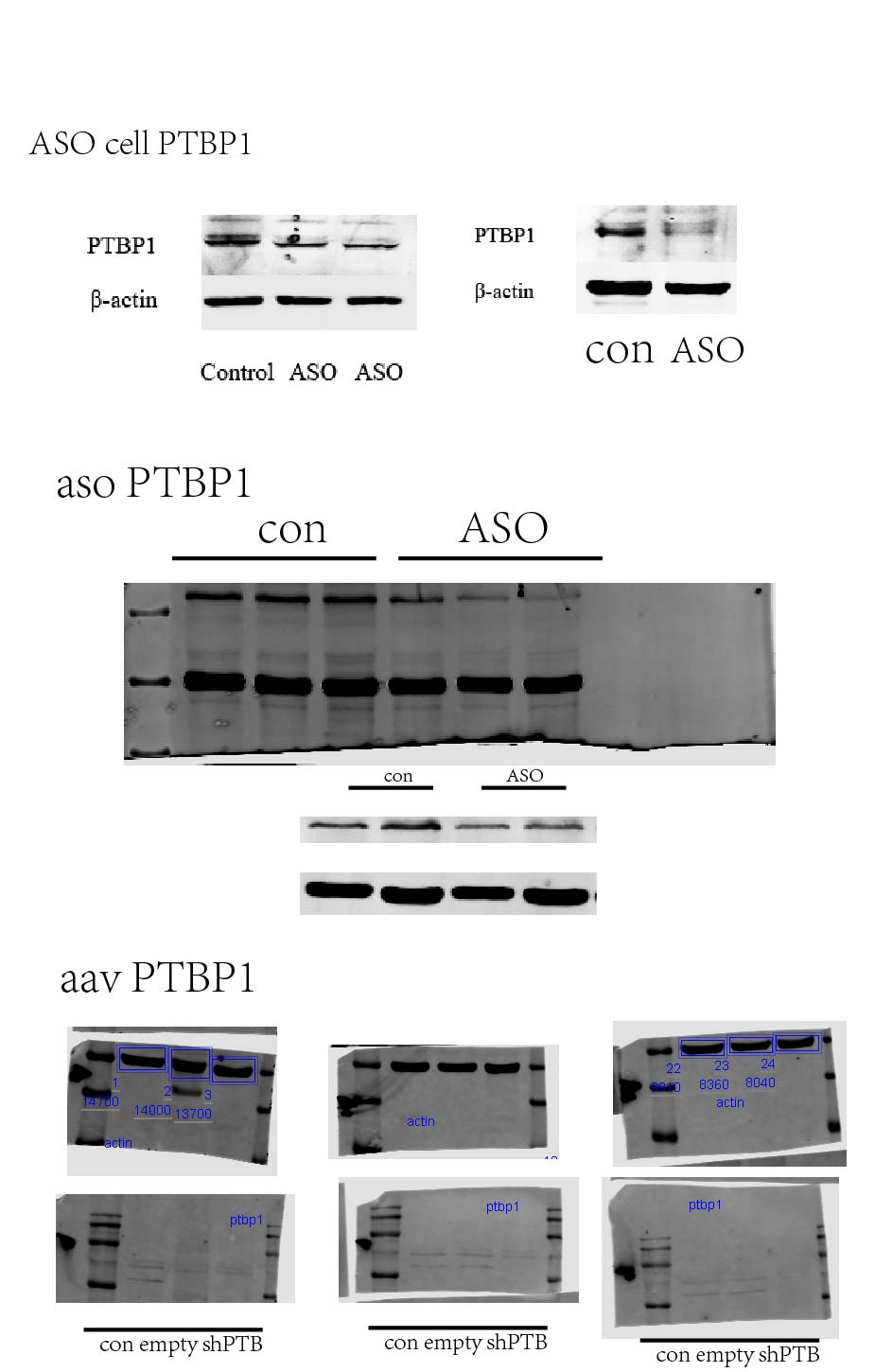

Supplement: Supplementary file 2 [file Image_1.JPEG]

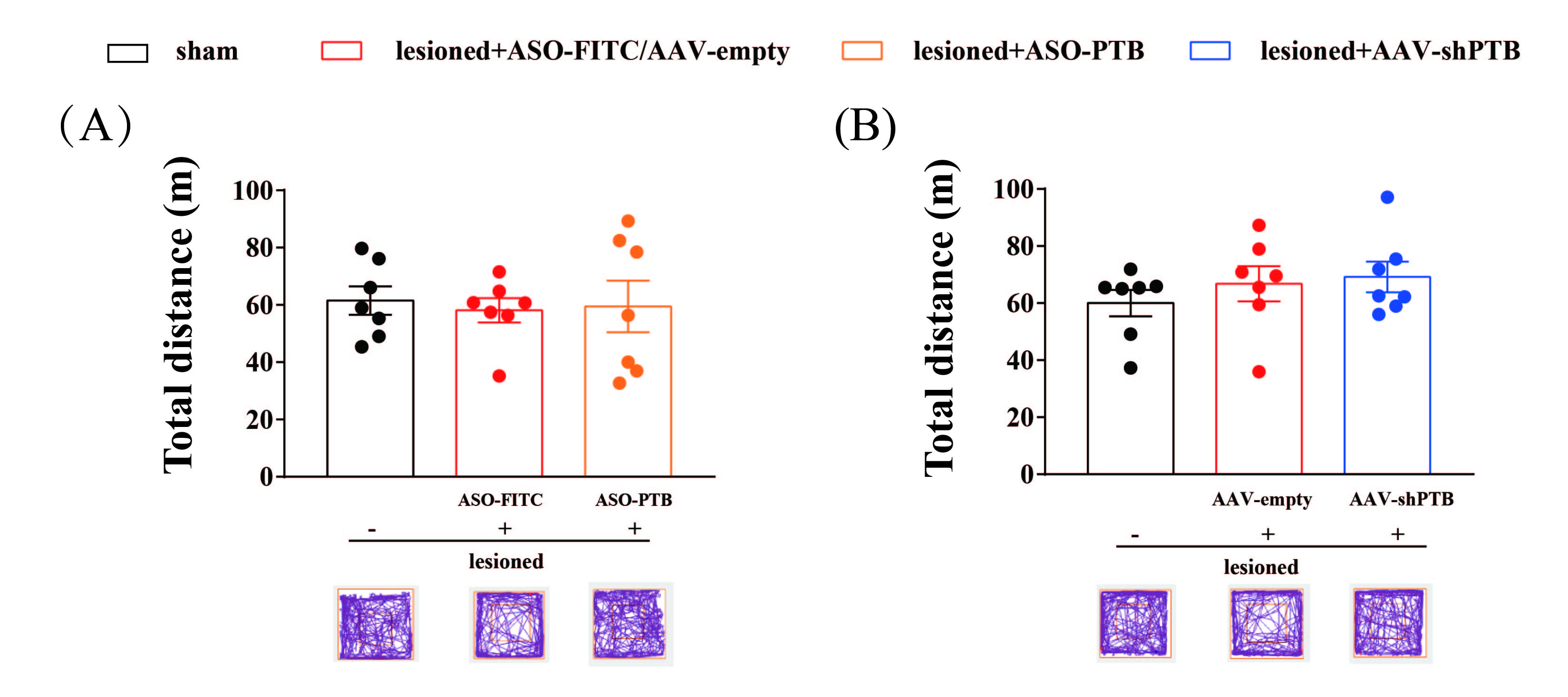

Supplement: Supplementary file 3 [file Image_2.JPEG]
